# Air-Jet Spun Corn Zein Nanofibers and Thin Films with Topical Drug for Medical Applications

**DOI:** 10.3390/ijms21165780

**Published:** 2020-08-12

**Authors:** Christopher R. Gough, Kristen Bessette, Ye Xue, Xiaoyang Mou, Xiao Hu

**Affiliations:** 1 Department of Physics and Astronomy, Rowan University, Glassboro, NJ 08028, USA; goughc2@students.rowan.edu (C.R.G.); bessettek1@students.rowan.edu (K.B.); xuey5@rowan.edu (Y.X.); 2 Department of Biomedical Engineering, Rowan University, Glassboro, NJ 08028, USA; 3 Department of Chemistry and Biochemistry, Rowan University, Glassboro, NJ 08028, USA; moux@rowan.edu; 4 Department of Molecular and Cellular Biosciences, Rowan University, Glassboro, NJ 08028, USA

**Keywords:** corn zein protein, composite material, drug delivery, air jet spinning, sodium citrate, nanofiber, film

## Abstract

Diabetic patients are especially susceptible to chronic wounds of the skin, which can lead to serious complications. Sodium citrate is one potential therapeutic molecule for the topical treatment of diabetic ulcers, but its viability requires the assistance of a biomaterial matrix. In this study, nanofibers and thin films fabricated from natural corn zein protein are explored as a drug delivery vehicle for the topical drug delivery of sodium citrate. Corn zein is cheap and abundant in nature, and easily extracted with high purity, while nanofibers are frequently cited as ideal drug carriers due to their high surface area and high porosity. To further reduce costs, the 1-D nanofibers in this study were fabricated through an air jet-spinning method rather than the conventional electrospinning method. Thin films were also created as a comparative 2-D material. Corn zein composite nanofibers and thin films with different concentration of sodium citrate (1–30%) were analyzed through FTIR, DSC, TGA, and SEM. Results reveal that nanofibers are a much more effective vehicle than films, with the ability to interact with sodium citrate. Thermal analysis results show a stable material with low degradation, while FTIR reveals strong control over the protein secondary structures and hold of citrate. These tunable properties and morphologies allow the fibers to provide a sustained release of citrate and then revert to their structure prior to citrate loading. A statistical analysis via *t*-test confirmed a significant difference between fiber and film drug release. A biocompatibility study also confirms that cells are much more tolerant of the porous nanofiber structure than the nonporous protein films, and lower percentages of sodium citrate (1–5%) were outperformed to higher percentages (15–30%). This study demonstrated that protein-based nanofiber materials have high potential as vehicles for the delivery of topical diabetic drugs.

## 1. Introduction

Over 10% of the United States was affected by diabetes in 2018, across all age groups [1]. Of particular concern for diabetic patients is chronic ulcers on the skin. If left untreated, these wounds can lead to infections and eventually the need for amputation. Sodium citrate is a topical drug [2] and can minimize diabetic induced damages. Sodium citrate acts as a buffer in order to neutralize the pH of the skin in patients with wounds, promoting faster healing. It is also used as a preservative to prevent invasion of microbial agents [3]. Citrate has received attention recently for its ability to better curate the immune response during drug delivery [4,5,6]. Among diabetes research specifically, sodium citrate has received attention for its ability to correct magnesium deficiency [7].

Topical delivery of dermatological drugs including sodium citrate offers many advantages over systemic delivery routes such as the requirement for lower doses and reduced off-target effects. The development of drug delivery vehicles such as micelles [3,8], liposomes [9,10], nanoparticles [11,12], nanofibers [13], and thin films [14], has allowed for long-term, controlled release of topical drugs when these systems are placed on the skin, increasing patient compliance and efficacy of the treatment. 1-D nanofibers and 2-D thin films are often easy to manufacture at a large scale and are able to efficiently target the site of injury, which improves the onset of drug action, reduces the dose frequency, eliminates side effects, and enhances drug efficacy [14]. Nanofibers are promising materials for dermatological drug delivery with notable mechanical properties, low density, high porosity, and large surface area, which enhances drug loading [15]. While, thin films provide a fast onset of action, reduced dose frequency, bioavailability, minimized drug loss, and rapid drug release [14].

Corn zein is the major storage protein found in corn, comprising about 80% of the total protein content in corn [16,17,18,19,20,21,22]. Within the medical and pharmaceutical fields, corn zein is widely used in a variety of biomaterials either by itself or as part of a composite. Several studies have shown the potential of electrospun corn zein as an effective substrate for cell culture [23,24,25,26]. Some of the most successful applications of corn zein in these industries has been in the form of fibers and thin films [27,28]. Corn zein also exhibits good biocompatibility and biodegradability, giving it great interest in topical drug delivery applications [23,29]. Biomaterials fabricated from corn zein also show antioxidative activity which prevents damage to nearby cells [30,31].

Corn zein is a unique material for nanofibers due to its high tensile strength, flexibility, and toughness in films, [32,33,34,35,36], nanofibers [24], and composites [37]. Fabrication of the fibers is also made easy by zein’s hydrophobicity and insolubility in water. One of the most common methods of fabricating nanofibers is electrospinning. While electrospinning is efficient and can easily tailor the microstructure of the fibers with repeatability, it also requires a high voltage source that limits the overall efficiency of the process due to dielectric constants [38]. Due to these problems, air jet-spinning was utilized as an alternative nanofiber fabrication method. Air jet-spinning, also called solution blowing, allows for the formation of a large volume of nanofibers from a polymer solution with high output efficiency [39]. Utilizing an air jet-spinning method to produce nanofibers is economical, simple to reproduce, and has a higher production rate than electrospinning [40]. Instead of a high voltage source, air-spinning uses compressed air to shear the polymer into fibers. In this method, the polymer solution is attached to a concentric nozzle spray gun and the high-speed air produced by an air compressor is used to stretch the polymer into the air as strands of nanofibers [39,40,41]. The high shear produced in this process causes the solvent in the solution to evaporate, leaving solid nanofibers that are deposited on a collection surface [39,41].

In this study, corn zein nanofibers and thin protein films were made with sodium citrate incorporated into the zein protein solution with different concentrations (1–30 wt%) prior to spraying or casting. By incorporating sodium citrate into zein fibers and films, a method for targeted and controlled delivery of sodium citrate to specific areas of the skin affected by diabetic ulcers is possible [42,43,44]. To see the effect of sodium citrate on corn zein-derived vehicles, materials were characterized using FTIR, DSC, TGA, and SEM. Additionally, a cell study was used to test the biocompatibility of the zein materials and understand the release effects of the sodium citrate drugs. A time-based drug release study was also performed to quantify the release of the drug and understand its release profile from zein fibers and films. Results demonstrated that corn zein nanofibers offer a better delivery vehicle compared to the films, since sodium citrate can integrate with the secondary protein structure of the fibers, shifting them from a random helical structure into a more ordered alpha helical structure. After releasing the sodium citrate, the protein structure returned to its original random helical structure, indicating a controlled, reversible transition that makes corn zein nanofibers a strong candidate for topical drug delivery.

## 2. Results and Discussion

### 2.1. Structural Characterization

**Figure 1:**
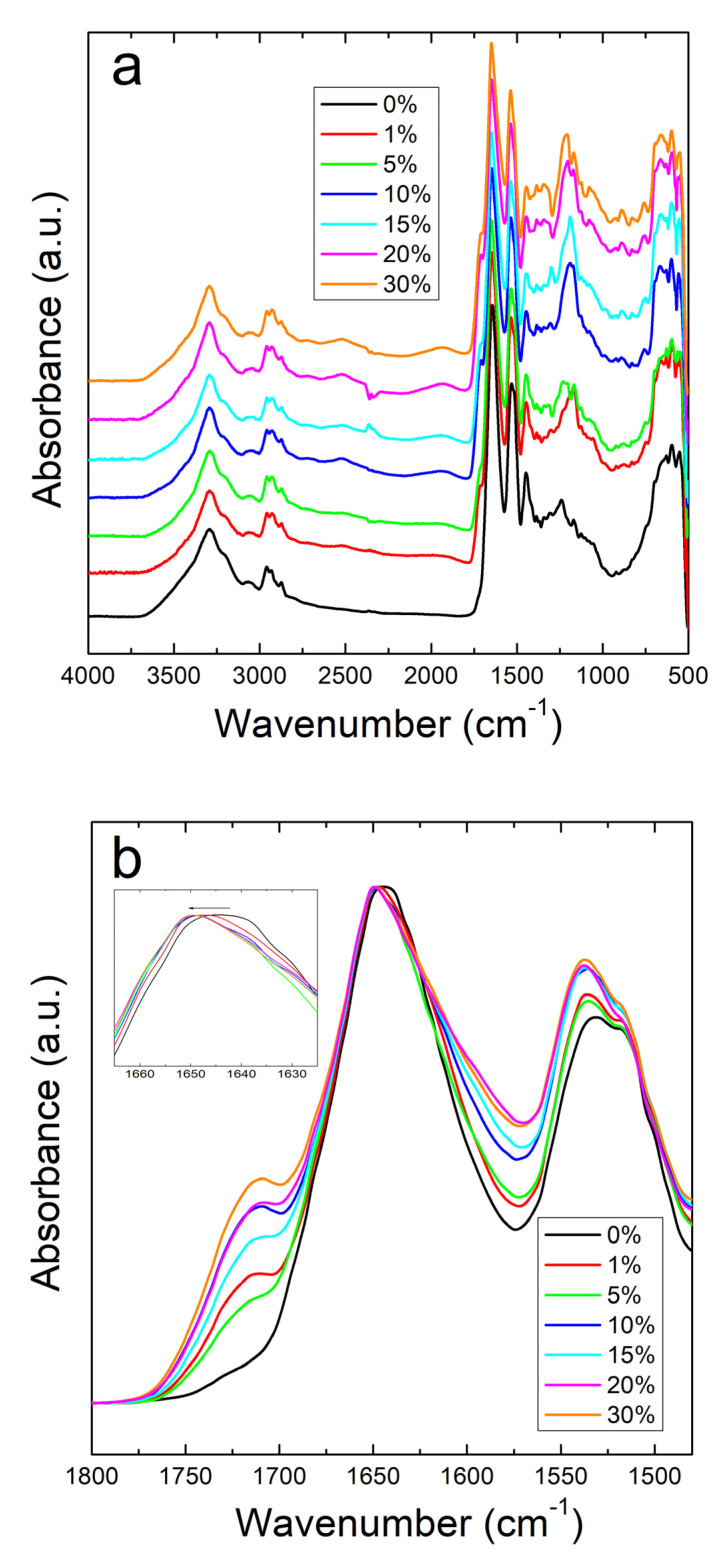
(**a**) FTIR absorbance spectrum for corn zein nanofibers with and without embedded sodium citrate. (**b**) Amide I and II regions of fibers: unloaded fibers show a peak at 1640 cm^−1^ indicative of random helical structure while fibers loaded with sodium citrate show a shift to alpha helical structure at 1650 cm^−1^ (see insert figure).

The structural properties of corn zein with sodium citrate were investigated by using Fourier Transform Infrared Spectroscopy (FTIR). A comparative study was performed by FTIR on both corn zein fibers and thin protein films, with different percentage of sodium citrate (0%, 1%, 5%, 10%, 15%, 30%). Figure 1a shows the full absorbance spectrum of the corn zein nanofibers. To better understand the secondary protein structure, Figure 1b is also included to highlight the Amide I and II regions. In the Amide I region specifically, the pure corn zein fibers show a peak located at 1640 cm^−1^, which is indicative of a protein network primarily consisting of random coils [45,46,47]. Upon addition of sodium citrate, this peak shifts to 1650 cm^−1^, representative of an alpha-helical protein structure. This shows that zein proteins in 1D fibers can interact with sodium citrate molecules through hydrogen bonds or charge–charge interactions in order to shift into a more ordered protein structure. This interaction of the drugs with the fibers is further supported by comparing the Amide II region of nanofibers (Figure 1a). Here, there is a slight shift from 1531 to 1538 cm^−1^ in fibers due to the denser structure of alpha helices, causing more side chains to become exposed on the outside of the protein backbones. There is also an increased absorbance in the drug loaded nanofiber samples compared to the pure nanofiber sample without sodium citrate.

**Figure 2:**
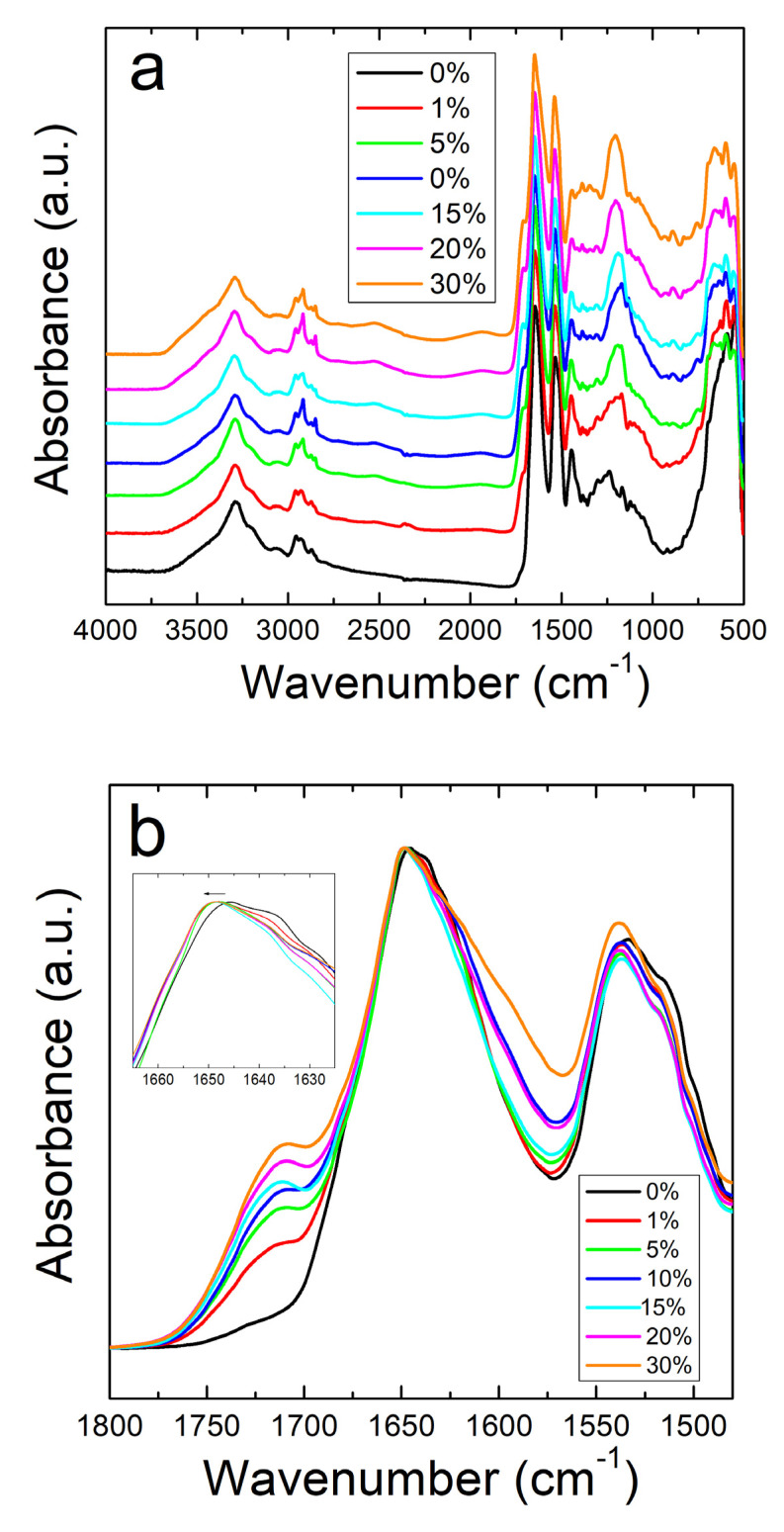
(**a**) FTIR spectrum for corn zein films with and without sodium citrate embedded. (**b**) Amide I and II regions for films: unlike the nanofibers, there is an insignificant shift in absorbance peak when sodium citrate drug is embedded.

Unlike the nanofibers, zein thin protein films do not show the same interactive behavior with sodium citrate. Figure 2a shows the full absorbance spectrum of the zein protein films with and without sodium citrate. The Amide I and II regions are better shown in Figure 2b. Protein films without sodium citrate show a peak at 1647 cm^−1^, representative of a helix dominated structure [48]. Although there is no clear trend, there is a decrease in the shoulder at 1638 cm^−1^ with the addition of sodium citrate, corresponding to a decrease in random coil structure. This could also be related to zein’s tendency to decrease in alpha helical structure after exposure to and evaporation from solvents [49]. Interestingly, the addition of this drug introduces a peak at 1386 cm^−1^ in the protein films, but not in the fibers. Absorbance peaks in this area are associated with the symmetric stretching of –COO bonds [50], which is likely a result of residual formic acid residing inside the 2D films. In the case of nanofibers, the large majority of formic acid is evaporated during 1D fiber spinning, with the remainder removed during drying, thus preventing the appearance of this peak in the FTIR analysis of the fibers.

All samples with sodium citrate showed a shoulder at 1710 cm^−1^. This shoulder increases in intensity with an increase in wt% of sodium citrate and is missing in nanofibers or films without the drug (Figure 1b and Figure 2b). It can also be speculated that the shoulder at 1710 cm^−1^ is a result of C=O stretched from hydrogen bonds between protein chains and sodium citrate molecules [51]. The addition of this drug also introduces a peak at 1572 cm^−1^ due to the interaction of side chains in the drug and antisymmetric stretching of -COO bonds [50]. Absorbance peaks in the spectrum that are associated with water also occur commonly in the samples with a large amount of sodium citrate, with a broad peak appearing in the 2000–1800 cm^−1^ range.

### 2.2. Thermal Analysis by DSC

**Figure 3:**
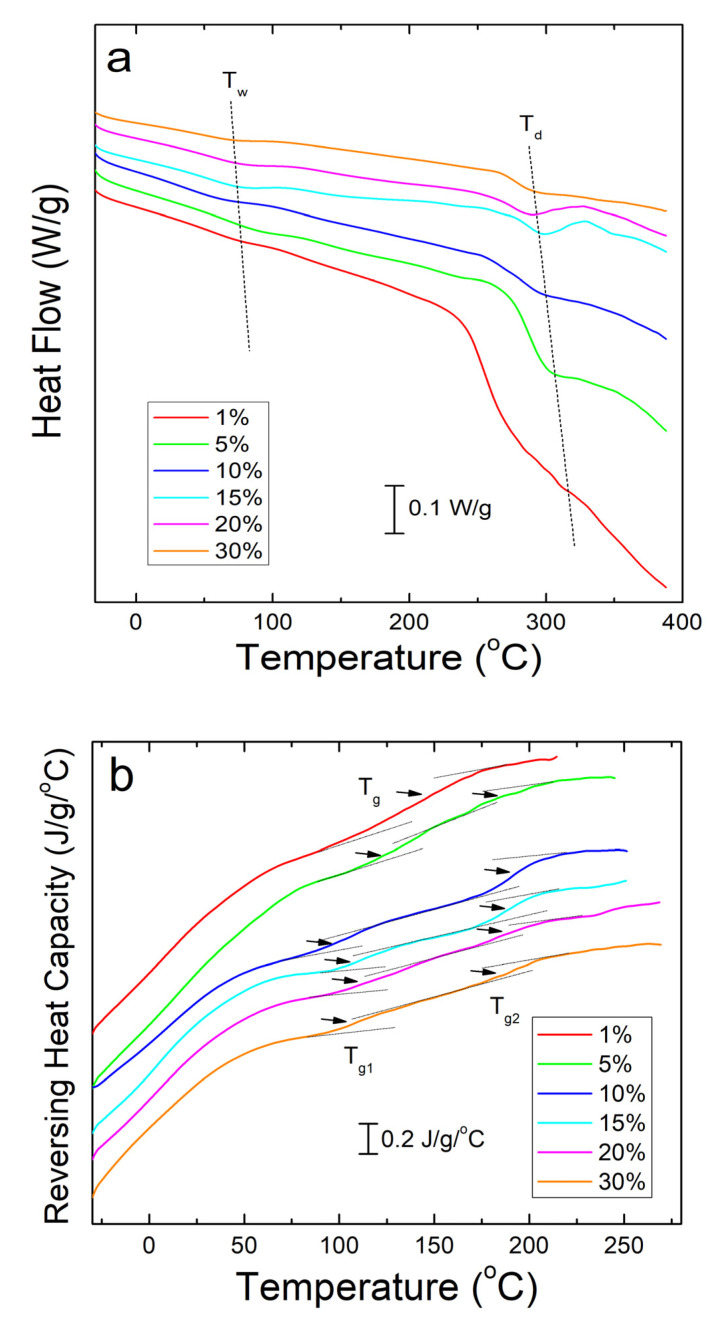
(**a**) DSC total heat flow analysis of corn zein nanofibers embedded with various wt% of sodium citrate. There are drops in the heat flow at T_w_ due to water evaporation and T_d_ due to thermal degradation. (**b**) Reversing heat capacity of corn zein nanofibers embedded with sodium citrate. Two glass transition temperatures (T_g1_ and T_g2_) can be found for the fiber samples with the drug.

Differential scanning calorimetry (DSC) analysis of corn zein nanofibers embedded with sodium citrate drug reveals two key temperatures related to heat flow through the sample. Shown below in Figure 3a, these are T_w_ related to the evaporation of water from the samples and T_d_ related to degradation of the sodium citrate embedded protein fiber samples themselves (Table 1). The samples are stable at temperatures well above physiological conditions, rendering them safe for the topical delivery of drugs.

To see the glass transition temperatures at a higher resolution, the reversing heat capacity of the fiber samples was also examined (Figure 3b). Using this method, most composite samples showed two glass transition temperatures (Table 1), although the initial temperature, T_g1_, is a much broader peak that becomes more defined at higher wt% of sodium citrate. At 1 wt% sodium citrate, this first transition temperature is indistinguishable. This implies that T_g1_ is related to sodium citrates interaction with the corn zein. T_g2_ is a much more defined peak at temperatures that correlate well with the heat flow graphs in Figure 3a. At temperatures surpassing T_g2_ there is a clear breakdown of the composites in Figure 3b. The high T_g1_ (>100 °C) and narrow T_g1_ steps seen in this DSC analysis are ideal for a drug carrying composite as they indicate the corn zein and the sodium citrate form a stable composite with strong interactions that is thermally stable at high temperatures.

**Figure 4:**
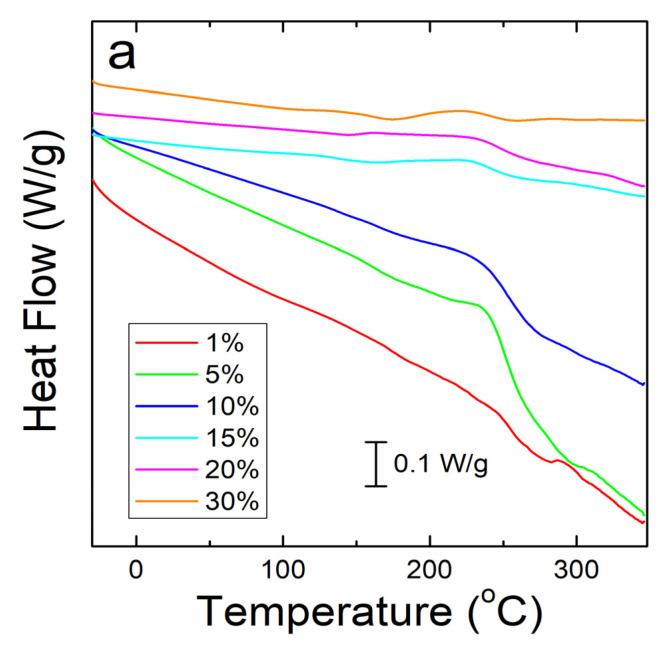

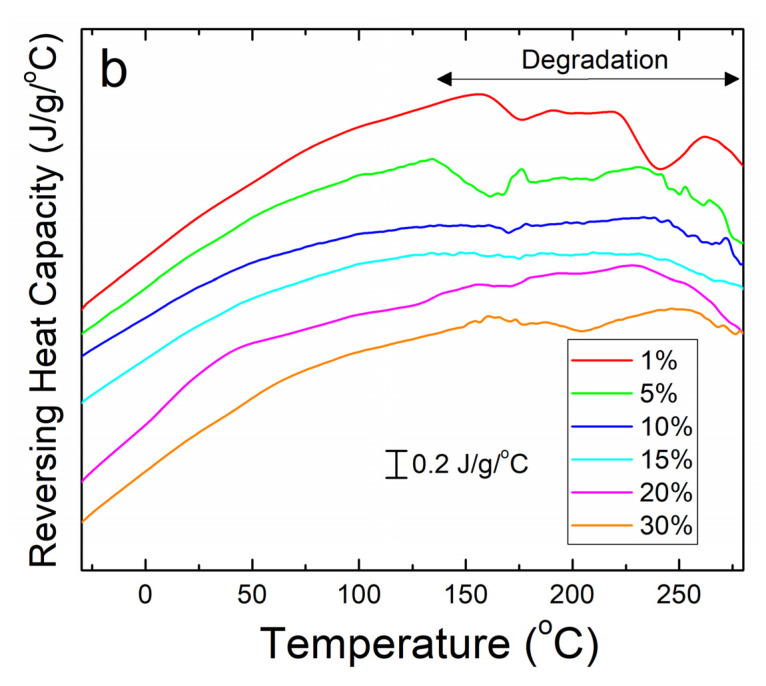
(**a**) Heat flow of zein protein films embedded with sodium citrate drug. All film samples degrade earlier than the nanofiber samples. (**b**) Reversing heat capacity of zein protein films embedded with sodium citrate drug. Starting around 150 °C, all samples start to show degradation picked up by the DSC as noise.

A similar analysis was done on corn zein protein films embedded with sodium citrate drug. Although there is less numerical data to extract from these analyses, there is a visible trend seen in Figure 4a. As more sodium citrate is added to the protein films, there is less degradation, indicating that the addition of the drug helps thermally stabilize the films. However, all protein film samples still degrade at a much lower temperature than the nanofiber samples. This result was predicted based on the FTIR analysis, which shows less intermolecular interactions between the protein structure and sodium citrate molecules in the films to stabilize the composite. Protein films start major degradation by 220 °C while the nanofibers do not see sample degradation until at least 250 °C. Even under the higher resolution that reversing heat capacity provides (Figure 4b), the only visible trend is in the thermal stabilization and a smaller noise presence with an increase in weight percentage of sodium citrate in the sample. However, all protein films clearly start to degrade around 150 °C (Figure 4b).

### 2.3. Thermal Gravitational Analysis (TGA)

**Figure 5:**
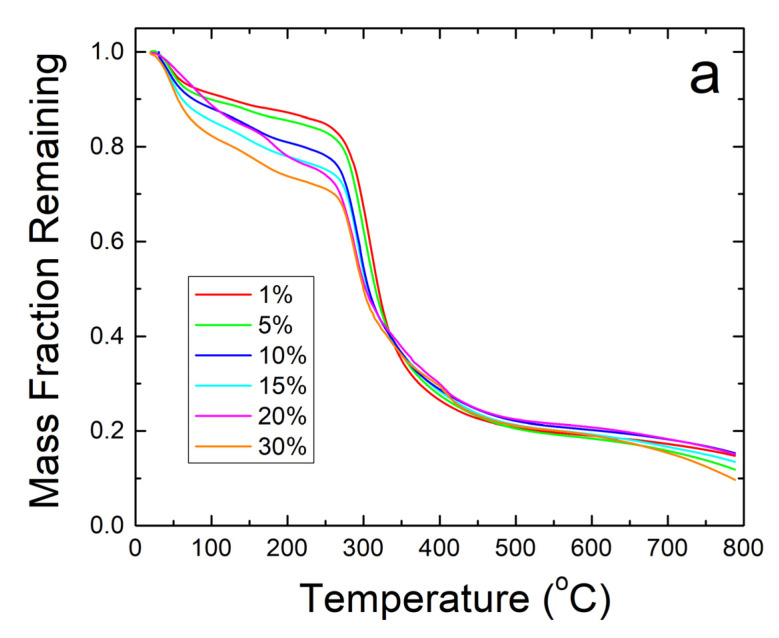

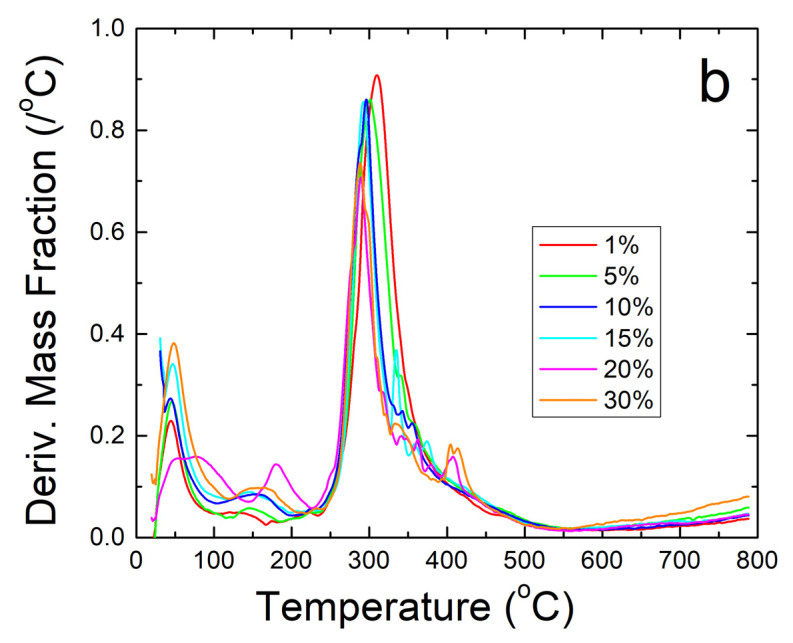
(**a**) TGA analysis of sodium citrate embedded corn zein nanofibers by weight fraction of sample remaining. As more sodium citrate is added, there is generally more degradation of the nanofibers as is most evident in the 200–300 °C region. (**b**) TGA analysis of sodium citrate embedded corn zein nanofibers by derivative of weight fraction remaining. Derivative with respect to temperature shows two peaks. The peak around 50 °C is likely attributed with loss of water. The main peak at 300 °C is attributed with zein and is well above physiological temperature for use as a topical drug delivery vehicle.

Further thermal analysis was performed on the samples using TGA. The steep decreases in fiber sample weight seen in Figure 5a correlate well with the T_w_ and T_d_ found earlier in the DSC analysis. The derivative of weight fraction, shown in Figure 5b, helps demonstrate this with the degradation rate. The initial peak around 50 °C correlates with T_w_ and shows the same trend where higher weight percentages of sodium citrate has higher bound water content and shift T_w_ lower. The largest peaks around 300–350 °C correlate with T_d_ where the sample degrades due to approaching the degradation temperature of zein, which is the dominant material in the composite.

**Figure 6:**
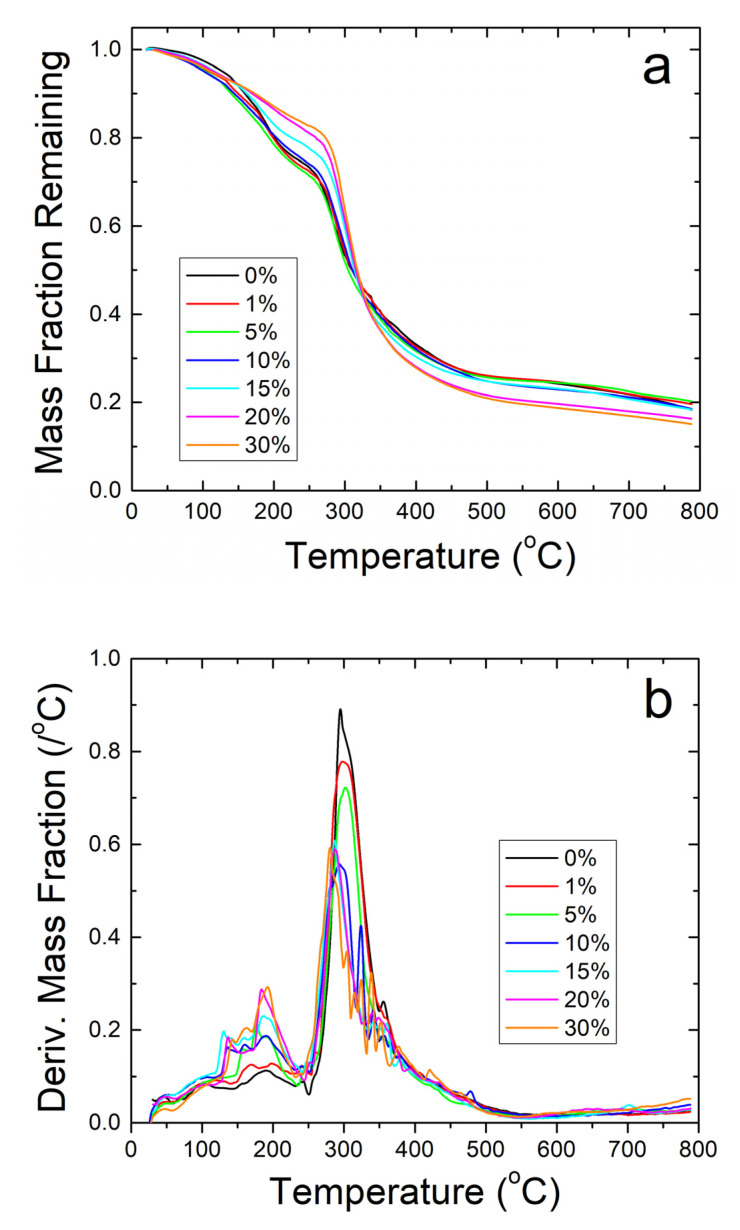
(**a**) TGA analysis on zein protein films with and without embedded sodium citrate. Samples retain a higher weight fraction than their nanofiber counterparts but degrade at a much lower temperature. (**b**) TGA analysis of protein films using derivative of remaining weight fraction. The large amount of noise shows thermal instability compared to the nanofiber samples.

TGA analysis on zein protein films also confirms the results about the thermal stability of the composites found during DSC analysis. Mainly, the analysis shows that the protein films cannot contain their protein structure at high temperatures. There is a large amount of degradation in all samples with a general trend to have less degradation at higher weight percentages of sodium citrate before 300 °C, as seen in Figure 6a. The high amount of noise peaks between 100 and 250 °C presented in Figure 6b also shows the low stability of the protein structure in the composite protein films. No major water peak was found near 75 °C for all film samples, similarly to Figure 4a. There is good correlation between Figure 4b and Figure 6b: both show that strong degradation begins around 150 °C in the zein-sodium citrate composite protein films. Based on the TGA analysis performed, sodium citrate may interact with the protein films in some way to help stabilize them given the higher remaining weight percent compared to the loaded samples, but the nanofibers are able to retain their protein structure over a much higher temperature than the protein films due to the interaction with its protein structure seen in the FTIR analysis.

### 2.4. Morphology Analysis

**Figure 7:**
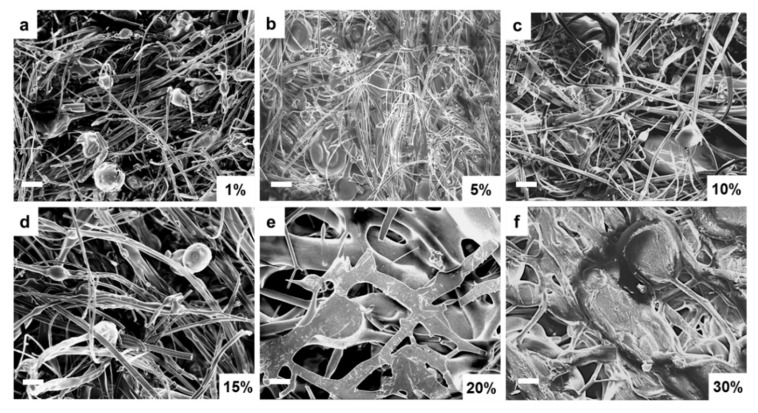
SEM images of sodium citrate embedded nanofibers at various weight percentages of sodium citrate ranging from 1% to 30%: (**a**) 1 wt%, (**b**) 5 wt%, (**c**) 10 wt%, (**d**) 15 wt%, (**e**) 20 wt%, and (**f**) 30 wt% sodium citrate embedded nanofibers. (bar size: 10 µm).

The above structural and thermal analysis studies clearly demonstrate that the unstable film samples are not suitable for long-term biomedical applications. Therefore, further research will focus on assessing whether fiber samples can be good candidates for biomedical research. Scanning electron microscopy (SEM) was first performed on corn zein fibers at various sodium citrate concentrations in order to reveal their morphology. Figure 7a–c contains low weight percentages (1–10 wt%) of sodium citrate. At these concentrations, the fibers are on the nanoscale, and their formed networks are highly porous, allowing for optimal drug transport. The sodium citrate is able to form a composite with the nanofibers at these lower weight percentages without losing the nanoscale architecture that makes nanofibers an appealing candidate for drug delivery such as their high porosity and surface area to volume ratio. There is not a noticeable structural difference between these figures. Although some droplets of sodium citrate are visible, they do not affect the morphology of the nanofibers significantly. Figure 7d–f contains 15–30 wt% sodium citrate; here the fibers are much larger than nanoscale, and there is very little porosity or availability for drug transport. At this point, the fibers may be too highly saturated with drug and lose their beneficial properties.

### 2.5. Drug Release Profile

**Figure 8:**
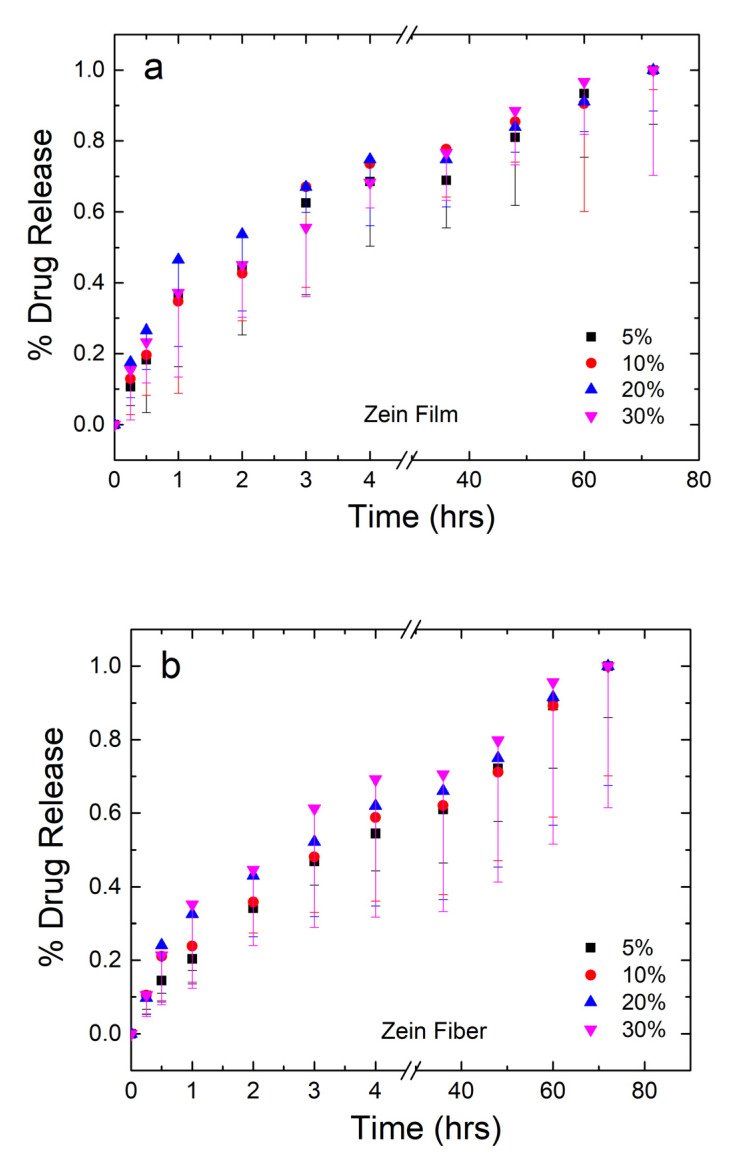
Normalized drug release profiles of corn zein films (**a**) and fibers (**b**) loaded with sodium citrate ranging from 5 to 30 wt% drug/zein with sampling occurring at 0.25–72 h.

The mass of drug released from the samples was analyzed and plotted in Figure 8a,b for the zein films and fibers, respectively. The amount of drug from each set of samples was averaged and normalized, where 100% is the mass released at the 72-h time point. For the plot in Figure 8a,b, error bars were added to represent the standard deviation and the average mass lost to degradation of the material themselves based on the mass lost in the 0% samples, respectively.

Both film and fiber samples see the majority of the drug released within the first 6 h. Film samples have a higher plateau ranging between 69% and 75% of drug released. From the 36–72 h points, the remaining drug is released from the samples. Before the plateau, 30% films saw the slowest release, but the reverse was true after the plateau. This may indicate the drug having some interaction with itself, where a large quantity of the drug is able to protect itself from dissolution. This interaction is likely lead by hydrogen bonds or charge–charge interactions since the molecule contains an alcohol and several carbonyl groups. Once a large enough amount of the drug has dissolved around the 75% mark, however, the drug is more susceptible to dissolution and goes into solution just as easily as the lower percentage samples.

Fiber samples plateau between 59% and 70% in the first 6 h, indicating that fibers have a slower, more controlled release of the drug. This can be explained by the large surface area-to-volume ratio of nanofibers, which make them desirable candidates for drug release in several other studies [52,53,54]. Additionally, as seen in the FTIR analysis, zein is able to interact with sodium citrate on a molecular level via hydrogen bonding in the fiber samples, but not the film samples. This interaction results in the slower release of sodium citrate in nanofiber samples compared to the film samples.

In order to confirm a statistically significant difference between the release of citrate from fibers and films, a paired two-sample *t*-test was performed for each citrate wt%. Table 2 summarizes the results of the *t*-tests performed in each sample group. For all groups, the T-statistic was compared to a T-critical of 1.812. From this analysis, the calculated T-statistic for each wt% group was 3.948, 3.363, 4.128, and 1.464 for 5, 10, 20, and 30 wt%, respectively. With 5%, 10%, and 20% having T-statistics higher than T-critical, the null hypothesis was rejected. From here, the *p* value was compared to the alpha-level to determine the significance. With *p* values of 0.003, 0.007, and 0.002 for the 5, 10, and 20 wt% samples all falling below a 0.05 alpha-level, it is concluded that all three of these groups have a statistically significant different release of citrate between their respective groups. Once drug loading reaches 30 wt%, however, the T-statistic falls to 1.464 which is below the T-critical of 1.812, resulting in a rejection of the null hypothesis for the 30 wt% group. Additionally, the 30 wt% group has a *p* value of 0.174, which is higher than the 0.05 alpha-level. Thus, at 30 wt% citrate, zein fibers and films do not have a statistically different release of citrate. This analysis aligns with qualitative data, in which 30 wt% fibers become brittle and lose their porosity, resulting in a loss of the valuable properties that make nanofibers an effective drug delivery vehicle. All four groups had a Pearson correlation coefficient very close to 1, showing very high linear correlation.

### 2.6. Cell Compatibility and Effect of Sodium Citrate Release

**Figure 9:**
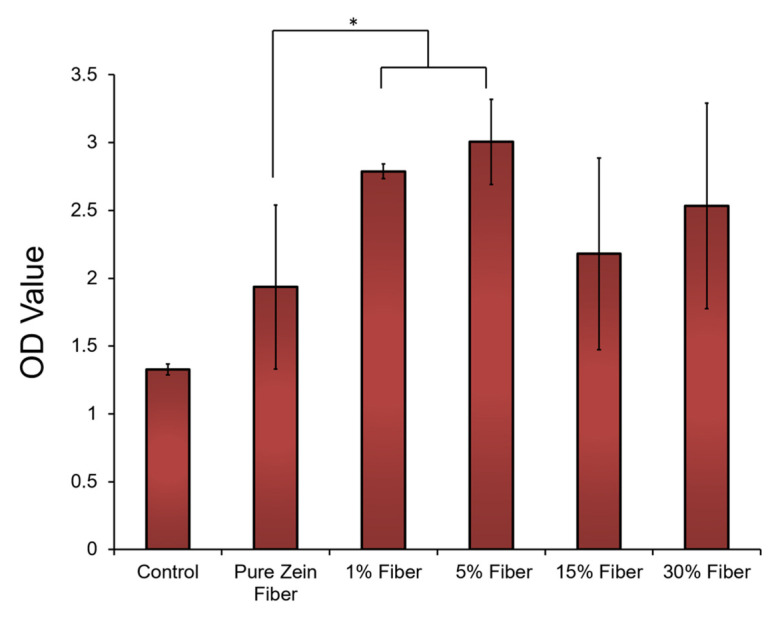
HEK293 (Human embryonic kidney) cells attached to and proliferated on zein fibers with different concentrations of sodium citrate. Cell density was assessed using MTT assay and plotted vs. blank substrate as control. The *t*-tests were performed between indicated groups (* *p* < 0.05).

Cell compatibility tests were performed to further demonstrate the potential of zein nanofibers for topical diabetes treatment applications. Human embryonic kidney cells (HEK293 cells) were cultured on the surface of zein fiber mat samples with 0–30% sodium citrate for 72 h (the blank substrates were used as the controls). After seeding the cells on the samples for 72 h, the morphology of the cells on all tested zein fiber materials remained stable, similarly to the cells on the blank substrate. Cell proliferation on the zein materials were then evaluated by cell numbers at 72 h using an MTT assay (Figure 9). After 72 h, compared with blank surface, zein fiber materials showed a significantly increased cell density, which indicated cell proliferation can be elevated by zein protein materials. In addition, at 72 h, the cell density on zein fiber samples increased with the increasing sodium citrate at a small concentration (<15%), indicating that these concentrations are suitable for biomedical applications. However, once the sodium citrate concentration reaches 15–30%, the cell density will decrease significantly, which indicates that the release of high concentrations of sodium citrate from the fiber material may prevent cell proliferation. According to SEM studies, this may also be due to the increase in fiber size to the microscale at a concentration of 15–30%, which provides less porosity for drug transport and reduces drug–cell interactions.

**Figure 10:**
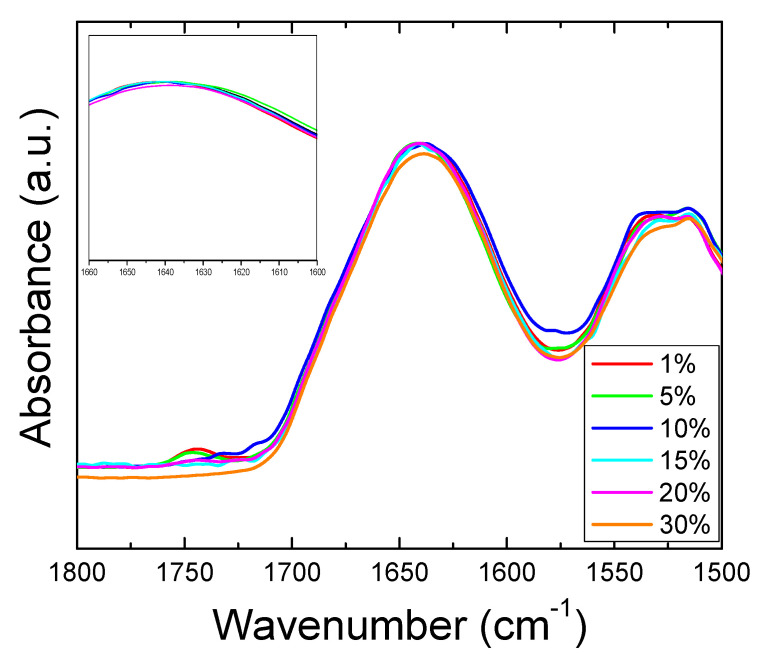
FTIR Amide I and II regions for fibers after release of sodium citrate. Absorbance peaks in Amide I all shifted back to 1640 cm^−1^ (insert figure), indicating that all zein fiber samples reverted back to the original random coil structure after the drug release.

In order to examine the effect of drug release on the structure of zein, after maintaining the fibers in a PBS solution environment for 72 h, the fibers were also re-analyzed by FTIR. As shown in Figure 10, the fibers revert back to their original random coil structure at 1640 cm^−1^. Additionally, the peak around 1720 cm^−1^ caused by C=O stretching between protein chains and sodium citrate hydrogen bonds disappeared completely. This reversible transition makes the corn zein nanofibers an ideal candidate for drug delivery of sodium citrate, since the drug stabilizes the fibers but does not permanently alter their structure on the molecular scale.

### 2.7. Mechanism

**Figure 11:**
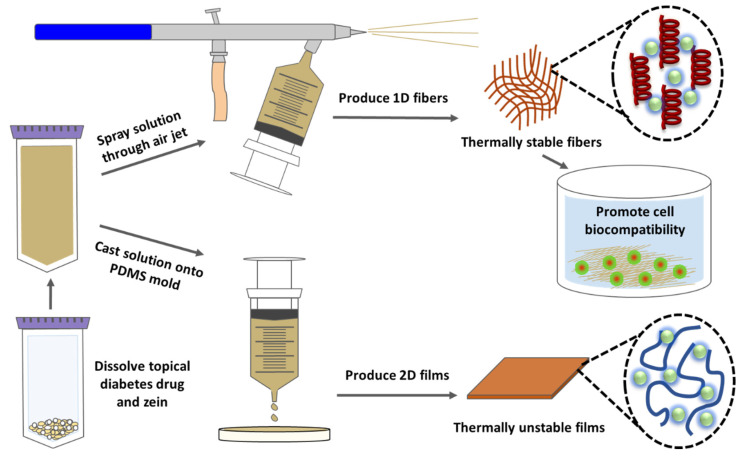
Mechanism of interaction between sodium citrate drug and corn zein nanofibers or protein films.

To explain the increased thermal stability of corn zein nanofibers compared with their protein film counterparts, it is likely that the sodium citrate interacts with the nanofibers in a way that it cannot with the protein films. Looking at the FTIR analysis, there is an interaction within the Amide I and II regions in the drug-embedded fibers. Upon addition of the drug, the protein structure of the composite shifts from a random helical structure into an alpha helical conformation. An alpha helical structure is stabilized by hydrogen bonding, which explains how the nanofibers are able to withstand high temperatures before degrading. Thermal analysis via TGA further confirms that the zein-citrate composites are stabilized with a reduction in degradation when more citrate is added to the samples. In the DSC analysis, this interaction can be seen in the steep step at T_g1_ that becomes more defined at higher wt% of citrate. Since this protein structure transition is not seen in the protein films, and they instead retain a random coil dominated structure, the film samples degrade at a much lower temperature than the nanofibers. Despite the zein–sodium citrate interaction, however, the nanofibers can revert back to their original structure after the drug has been released. This makes corn zein nanofibers an interesting candidate for drug delivery of sodium citrate; not only does the drug interact with the corn zein to stabilize it, but it does not permanently alter the protein structure of zein. This mechanism is illustrated below in Figure 11.

### 2.8. Reflection and Future Considerations

Some issues during the formation or characterization of materials occurred during this study. Specific to fabrication of nanofibers, the high ambient humidity of the atmosphere made air-spraying difficult. In order to overcome this, several samples of zein-citrate solutions were prepared and spun in sequential order on a day with low humidity; ideally, the humidity should be below 20% to allow the solvent to evaporate off during the spinning process. Note, however, that electrospinning is also hindered by high humidity [55]. Solutions could not be prepared in advance, as this would cause solutions to become too viscous to air-spray. A humidity-control box can be developed to implement in future experiments in order to try to minimize the effects of ambient humidity.

While mechanical integrity is an important aspect of biomaterials and their transition into the clinical setting, accurate mechanical properties were not able to be characterized in this study. Typically, mechanical testing on biomaterials is done in PBS or after soaking in PBS. Due to the immediate release of citrate from the samples, we found it to be difficult to find reliable, repeatable measurements from initial mechanical testing. Despite this, zein films and fibers were palpable, flexible, and easy to move throughout drug release testing.

In the present study, it was found that citrate did not have a strong impact on the absorption of the solution, making the standard UV-Vis spectra method for measuring release impossible for citrate. FTIR and pH probes were also used to try to quantify the release of citrate from the zein vehicles, but any changes in absorbance or pH were not significant enough to measure. Due to this, the best method to quantify release was to measure the mass caught by filter paper using an analytical balance, as described in Section 3.8. However, UV-Vis spectra were able to measure the release of several model drugs from zein fibers and films. We also tested the model drugs using the same mass method, and the results did not show a significant difference between the UV and the mass methods, confirming that the mass method is reliable for this study.

## 3. Materials and Methods

### 3.1. Preparation of Materials

Zein protein powder was a gift obtained from POET, LLC (Sioux Falls, SD, USA), with purity larger than 87%. ACS formic acid of Grade 98% was purchased from EMD Millipore Corporation (Burlington, MA, USA). Sodium citrate tribasic dihydrate of greater than 99% purity for molecular biology was obtained from Sigma-Aldrich (St. Louis, MO, USA). All three materials were used as-is from the supply company without further preparation.

### 3.2. Corn Zein Nanofibers

Nanofibers were fabricated by dissolving 5.3 g of corn zein into 10 mL of formic acid at room temperature with mixing using a benchtop vortexer (VWR International, Radnor, PA, USA). Prior to dissolving corn zein, sodium citrate was dissolved into formic acid at concentrations of 1%, 5%, 10%, 15%, 20%, and 30% (weight drug/weight zein) for samples containing the drug. The dissolved zein-sodium citrate solution was transferred to a disposable syringe (VWR International, Radnor, PA, USA) and attached to a Neo Siphon-Feed Dual-Action Airbrush from Anest Iwata-Medea, Inc. (Portland, OR, USA), via a plastic attachment made in-house from a piece of tubing. A DeWalt D55168 air compressor (Baltimore, MD, USA) was used to supply the airbrush with 100 psi of pressure, which provided shear to produce the fibers. The solution was sprayed into an aluminum foiled lined box approximately 1.5 m away and dried overnight in a vacuum oven (VWR International, Radnor, PA, USA) at 60 °C to evaporate the excess formic acid.

### 3.3. Corn Zein Films

In order to compare the potential of zein fibers as a drug carrier compared to other forms of zein materials, thin protein films were also casted from the zein protein solutions prepared in the same way as stated above. In total, 2 mL of each solution was casted into PDMS molds and dried at room temperature for 2 days. To remove excess formic acid, films were also placed in a vacuum oven at 60 °C overnight. The thickness of the films was about 20 µm.

### 3.4. Scanning Electron Microscopy (SEM)

The morphology of all samples was studied by Scanning Electron Microscopy (SEM) imaging using a LEO 1530 VP SEM (LEO Electron Microscopy Inc., Thornwood, NY, USA). For better conductivity, samples were sputter coated with gold for 15 s prior to imaging. Images were acquired with an EHT of 10 kV.

### 3.5. Fourier Transform Infrared Spectroscopy (FTIR)

Fourier Transform Infrared Spectroscopy (FTIR) analysis was performed on all samples using a Bruker Tensor 27 Fourier Transform Infrared Spectrometer (Billerica, MA, USA) with deuterated triglycine sulfate detector and constant nitrogen gas purging. A multiple reflection, horizontal MIRacle ATR attachment (Ge crystal from Pike Tech, Madison, WI, USA) assisted in acquiring the spectra, which ranged from 4000 to 400 cm^−1^ with 64 scans at a resolution of 4 cm^−1^ intervals. Readings were taken twice on each side of the sample to ensure homogeneity, but only one spectrum from each sample is shown to better highlight the trend of drug loading and de-loading on the samples. The ATR crystal was cleaned with methanol between samples, and 64 background scans were performed before any readings were taken.

### 3.6. Differential Scanning Calorimetry (DSC)

The thermal stability of samples was analyzed through temperature-modulated differential scanning calorimetry (TM-DSC) using a Q100 DSC (TA Instruments, New Castle, DE, USA) outfitted with a refrigerated cooling system. A total of 5 mg of each sample was loaded into aluminum pans and temperature measurements were taken from −40 to 400 °C at a heating rate of 2 °C/min with modulation period of 60 s and temperature amplitude of 0.318 °C. Dry nitrogen purge gas was used at a rate of 50 mL/min. Prior to measurement, the instrument was calibrated with respect to heat flow and temperature using an indium standard and with respect to heat capacity using aluminum and sapphire standards.

### 3.7. Thermal Gravitational Analysis (TGA)

A TA Instruments TGA model SDT-Q600 (New Castle, DE, USA) was used to measure changes in mass of thin film and nanofiber samples with increasing temperature. A nitrogen gas flow rate of 50 mL/min was used. The mass of the sample was recorded over a temperature range of 25–800 °C at a rate of 10 °C/min.

### 3.8. Drug Release Study

Small samples (≈6 mg) were prepared of the fibers and films, both at 5%, 10%, 20%, and 30% wt. drug/wt. zein in sets of five. Additional, 0 wt% samples of similar weight were prepared in triplicate in order to account for potential mass lost due to degradation of the fibers themselves in water. All samples were soaked in 200 μL of de-ionized water over 72 h. At set time points (0.25, 0.5, 1, 2, 3, 4, 5, 6, 12, 24, 36, 48, 60, and 72 h), the samples were moved to a new well of 200 μL of de-ionized water. Meanwhile, the water–drug solution left in the well was run through pre-weighed filter paper and then dried in a vacuum oven. Once dried and cooled long enough to register a consistent weight measurement, the drug content of the solution was recorded. Between measurements, the well plate was covered with parafilm to prevent water loss or contamination. A cumulative drug release was calculated as the sum of each of these time segments. The weight loss from the 0 wt% samples was recorded for an error factor. At the end of the study, samples were moved into empty wells and dried in a fume hood for 4 days, and their final weight were recorded.

### 3.9. Statistical Analysis

A statistical analysis comparing the release of citrate from the fibers and the films was completed first by using a paired two-sample *t*-test using the Excel software; the null hypothesis tested was that the mean drug released from the fibers was the same as the mean drug released from the films. The test was repeated for each wt% category (1%, 10%, 20%, and 30 wt%). For the 11 time points recorded, there were 10 degrees of freedom. A two-tailed alpha level of 0.05 was chosen for all samples. Statistical significance was tested by comparing T-statistics to T-critical and the *p*-value, the probability of the null hypothesis being true, to the alpha level. A Pearson correlation coefficient was also calculated to test for covariance between each wt% group.

### 3.10. Biocompatibility Study

HEK293 (Human embryonic kidney) cells from ATCC (American Type Culture Collection, Manassas, VA, USA) were grown in Dulbecco’s modified Eagle’s medium (HyClone, with 4.00 mM L-Glutamine and 4500 mg/L Glucose) supplemented with 10% fetal bovine serum (Life Technologies Inc., Carlsbad, CA, USA) and 100 U/mL Penicillin-Streptomycin (Thermo Fisher Scientific Inc., Waltham, MA, USA), in an atmosphere of 95% air, 5% carbon dioxide (CO_2_), at 37 °C. Cell culture was carried out according to NIH standard protocols. Before cell seeding, all samples were sterilized overnight under UV light. An equal number of cells was seeded on different zein fiber mat or film samples with blank substrates as the control samples. Cell numbers were acquired 72 h after seeding using a 3-[4,5-dimethylthiazole-2-yl]-2,5-diphenyltetrazolium bromide (MTT) assay.

## 4. Conclusions

This study revealed that corn zein nanofibers can be used as an effective carrier for the drug sodium citrate which can be used topically to treat diabetic ulcers. FTIR analysis revealed an interaction between sodium citrate and corn zein which shifted its protein structure from a random coil-dominated network to a much more stable alpha helical network. This interaction also results in a slower, more controlled release of sodium citrate from the fibers compared to the films. After drug release, the fibers returned to their original structure, showing a reversible transition that did not occur in zein protein films embedded with the drug. At the same time, the addition of the drug seems to minimally affect the thermal stability of the nanofibers as is indicated by DSC and TGA analysis. TGA shows a trend where more drug creates more degradation at high temperature (above 300 °C) and there seems to be a limit to the weight percent of drug that can be incorporated before the nanofibers lose their effectiveness as drug delivery vehicles. Cell proliferation studies have also confirmed that composite fibers with less sodium citrate (e.g., 5%) are more suitable for biomedical applications. This limitation is also evident in the SEM micrographs shown in this study. Regardless, zein nanofibers are a promising drug carrier that have potential to be used with other topical drugs. The unique tunable properties of biomaterials also provide a means to improve the current system by modifying the physiochemistry of the material to cater to specific applications. We envision combining this system with a temperature sensor in order to detect the swelling of ulcers, which can then alert the patient to administer a dosage of citrate in the future.

## Figures and Tables

**Table 1:** Critical temperatures associated with sodium citrate embedded corn zein fibers at various wt% of the drug.

Sodium Citrate in Sample (wt%)	T_w_ (°C)	T_d_ (°C)	T_g1_ (°C)	T_g2_ (°C)
1	81	311	139	N/A
5	83	305	128	189
10	73	297	104	190
15	78	297	109	188
20	75	290	116	187
30	69	289	105	186

All temperature values have an error bar within ±0.5 °C; T_w_ and T_d_ data are obtained from DSC total heat flow, and T_g1_ and T_g2_ data are obtained from reversing heat capacity.

**Table 2:** Statistical values obtained from a paired two sample *t*-test between fibers and films at each citrate wt% with an alpha level of 0.05. T-critical for all samples was 1.812.

Citrate wt%	Pearson Correlation	T-Stat	*p* (T ≤ t)
5%	0.983	3.948	0.003
10%	0.977	3.363	0.007
20%	0.984	4.128	0.002
30%	0.993	1.464	0.174
